# Biomarkers for Microglial Activation in Alzheimer's Disease

**DOI:** 10.4061/2011/939426

**Published:** 2011-11-01

**Authors:** Ronald Lautner, Niklas Mattsson, Michael Schöll, Kristin Augutis, Kaj Blennow, Bob Olsson, Henrik Zetterberg

**Affiliations:** ^1^Clinical Neurochemistry Laboratory, Department of Neurochemistry and Psychiatry, Institute of Neuroscience and Physiology, The Sahlgrenska Academy at University of Gothenburg, 43180 Mölndal, Sweden; ^2^Division of Alzheimer Neurobiology, Department of Neurobiology, Care Sciences and Society, Karolinska Institute, 17177 Stockholm, Sweden

## Abstract

Intensive research over the last decades has provided increasing evidence for neuroinflammation as an integral part in the pathogenesis of neurodegenerative diseases such as Alzheimer's disease (AD). Inflammatory responses in the central nervous system (CNS) are initiated by activated microglia, representing the first line of the innate immune defence of the brain. Therefore, biochemical markers of microglial activation may help us understand the underlying mechanisms of neuroinflammation in AD as well as the double-sided qualities of microglia, namely, neuroprotection and neurotoxicity. In this paper we summarize candidate biomarkers of microglial activation in AD along with a survey of recent neuroimaging techniques.

## 1. Introduction

### 1.1. Microglia in the Healthy Brain

The central nervous system (CNS) has long been regarded as an immune privileged organ, entirely separated from the peripheral immune system. However, this concept has been fundamentally revised in recent years, as more evidence about the existence and the function of the innate immune defence of the brain has become known [1]. Microglia play an outstanding role in this context as these cells constitute the first line of defence against noxious agents in the brain. They derive from peripheral macrophages and display a ramified morphology in the resting state. It is believed that their function is to constantly scan the surrounding microenvironment in order to detect possible changes in the extracellular homeostasis of the brain that might be harmful to neurones. Upon activation, microglia proliferate migrate to the site of the lesion and undergo a drastic change in morphology, obtaining phagocytic abilities and releasing proinflammatory cytokines. The main purpose of this process is to remove cellular debris and finally to restore homeostasis in the extracellular microenvironment of the brain, thus protecting neuronal tissue from collateral damage [2].

### 1.2. Microglial Activation in Alzheimer's Disease—Protective or Destructive?

Over the last decades, increasing evidence has suggested that neuroinflammation represents a crucial part in the pathogenesis of AD as well as in other neurodegenerative diseases [3]. It has been shown that activated microglia can be found in AD brains, surrounding extracellular deposits of *β*-amyloid, maintaining an inflammatory milieu by secreting pro-inflammatory cytokines [4, 5]. It has been hypothesized that this chronic inflammatory state contributes decisively to the progression of the disease, thus suggesting that activated microglia not only exert neuroprotective effects, but might also be detrimental for the survival of neuronal tissue. It is still unknown why and when, in the course of AD, microglia switch from being beneficial to becoming neurotoxic, but age-related disturbance of the physiological function and regulation of microglia (immunosenescence) has been suggested to play an important role in the AD pathogenesis [6]. Another question that remains to be answered is whether microglial activation occurs as a consequence of extracellular *β*-amyloid deposition in AD, or if it serves as a triggering factor for *β*-amyloid deposition in the initial stages of the disease. In animal models, it has been shown that microglia can be activated by extracellular *β*-amyloid [7]. However, the presence of activated microglia has been observed even before the onset of *β*-amyloid deposition [8].

## 2. Biochemical Markers

### 2.1. Use of Biochemical Markers of Microglial Activation

Biochemical markers of microglial activation that allow monitoring of the inflammatory state of the CNS, might be useful for the comprehension of the double-edged characteristics of microglia in AD. Furthermore, by investigating the relation between biomarker levels and disease progression, they could contribute to the understanding of underlying pathogenic mechanisms. In addition, comparing biochemical microglial markers with neuroimaging markers will help visualize the distribution of activated microglia throughout the brain. Taking a long view, these biomarkers could also be used to monitor therapy in studies of drugs that target microglial activation.

Cerebrospinal fluid (CSF) is in direct contact with the brain parenchyma and pathological processes in the CNS often lead to altered CSF levels of specific analytes, which is the basis for using CSF biomarkers in research and clinical neurology. During the last two decades, it has become clear that several core neuropathological hallmarks of AD may be monitored in CSF, with low A*β*42 levels secondary to amyloid pathology, and high tau and phospho-tau levels, secondary to axonal degeneration and tangle pathology, respectively [9]. CSF is also a possible source for markers of AD-related microglial activity. A number of biochemical markers of microglial activation have been described and investigated, not only in AD, but also in other diseases which involve neuroinflammation and/or activation of peripheral cells belonging to the macrophage lineage.

### 2.2. Chitotriosidase

Chitotriosidase is an enzyme that exerts chitinolytic activity without having any known physiological function in humans, as inherited enzyme deficiency remains asymptomatic [10]. It appears as a product of activated mononuclear cells and functions as a marker for lipid-laden macrophages in peripheral blood, which can be used for diagnosing and therapeutic monitoring of lysosomal storage disorders such as Gaucher's disease [11]. Chitotriosidase activity in the CSF of AD patients is significantly higher than in cognitively healthy controls, which strengthens the hypothesis that disease-related microglia markers may be monitored in AD. However, CSF chitotriosidase activity appears to be unsuitable as a diagnostic tool on its own, due to large overlap between patients and controls [12].

### 2.3. CCL18

CCL18, also known as PARC (pulmonary activation-regulated chemokine), is a macrophage-derived chemokine that mainly attracts T-cells [13] and, precisely as chitotriosidase, shows considerably elevated levels in blood from patients with Gaucher's disease, hence serving as a suitable surrogate marker for lipid-laden macrophages in lysosomal storage disorders [14]. The presence of CCL18 in the CSF was demonstrated in patients suffering from infectious meningitis [15]. Furthermore, the expression of CCL18 in the CNS has been elucidated in relation to traumatic brain injury and cerebral tumours [16] as well as to myelin-laden macrophages in and around demyelinating lesions in multiple sclerosis [17], suggesting an anti-inflammatory nature. In the previously mentioned study, CCL18 was not detected in the CSF of AD patients [12].

### 2.4. YKL-40

YKL-40 is a member of the same protein family as chitotriosidase (family 18 glycosyl hydrolases) and is highly similar in structure to chitotriosidase, but lacks chitinolytic activity as a result of a critical amino acid substitution. Its biological function is widely unknown, but a role in inflammation and tissue remodelling has been suggested [18]. It is released by activated macrophages and elevated concentrations have been measured in the CSF of AD patients [19]. However, the difference in YKL-40 between patients and controls in the study cited above was relatively small, with a large overlap between groups, and an independent smaller study could not reproduce the group difference [12], suggesting that alterations of CSF YKL-40 in AD are too small for this biomarker to be of diagnostic usability. However, more studies are needed to determine the ultimate value of YKL-40 as a biomarker for AD.

### 2.5. CCL2

CCL2, also known as MCP-1 (monocyte chemoattractant protein 1), is a chemokine, measurable in CSF and represents a microglial secretion factor that appears to play an important role in macrophage migration [20]. Elevated levels of CCL2 in the CSF of AD patients have been described [21, 22], but a recent study failed to reproduce this [12].

### 2.6. CD14

CD14 is a surface protein, mainly expressed by macrophages and acts as a cofactor for toll-like receptors (TLRs), which are essential for the recognition of pathogens by the innate immune system of the brain [23]. In AD, it has been shown that microglial TLRs and CD14 are involved in the inflammatory reaction surrounding *β*-amyloid deposits [24]. In animal models, the deletion of CD14 resulted in a change of the inflammatory response, decreasing the number of activated microglia and the amount of *β*-amyloid plaques [25]. One study examined cell adhesion molecules in purified monocytes from the peripheral blood of patients with AD and cognitively normal controls and found decreased ratios of monocytic ICAM-3/CD14 and P-selectin/CD14 in AD [26]. These results suggest that the expression of monocytic cell adhesion molecules is decreased in AD. Another interpretation is that CD14 expression *per se* is increased. In fact, elevated levels of a soluble form of CD14 were recently seen in CSF from AD and Parkinson's disease (PD) patients compared with healthy controls [27]. The lack of longitudinal data in these studies prevents from drawing any conclusion on whether upregulation of CD14 is beneficial or detrimental in the disease process. 

### 2.7. Neopterin

Neopterin is a degradation product deriving from the purine nucleotide guanosine triphosphate (GTP). It is secreted by macrophages upon activation and may stimulate the production of reactive oxygen species (ROS) [28, 29]. It can be utilized as an indicator for immune system activation, showing elevated levels in several infectious diseases, autoimmune disorders, and malignant tumours as well as following allograft rejection [30]. Neopterin has even been measured in the CSF of AD patients, however without significant differences compared to controls [31].

## 3. Neuroimaging

*In vivo *visualization of microglial activation has become possible with the development and introduction of molecular imaging ligands (tracers) for use with positron emission tomography (PET) or single-photon emission tomography (SPECT). Most so far available imaging ligands make use of their high affinity to the peripheral benzodiazepine binding site (PBBS/PBR), also called translocator protein (TSPO), a receptor located in the outer membrane of mitochondria. Its upregulation within the CNS has been shown to reflect neuroinflammatory processes, mainly due to the activation of microglia [32, 33]. The most extensively employed PET imaging ligand to study microglial activation in various brain diseases both in humans and transgenic animal models is the ^11^C-labeled isoquinoline (*R*)-PK11195 (1-[2-chlorophenyl]-*N*-methyl-*N*-[1-methyl- propyl]-3-isoquinoline carboxamide) (PK11195), a specific ligand for the PBBS. An early PK11195 PET study that did not show any difference between AD patients and a group of controls suffered from several methodological issues [34]. Subsequent PET studies used the aforementioned more active *R*-enantiomer of PK11195 and applied different and more advanced quantification approaches to enhance tracer evaluation [35, 36].

One PK11195 PET study found increased binding levels in the entorhinal, temporoparietal, and cingulate cortex in a group of AD patients and one subject with mild cognitive impairment (MCI) as compared to normal individuals [37]. Several studies have combined PK11195 PET imaging with PET examinations using the fibrillar amyloid imaging ligand ^11^C-Pittsburgh Compound B (PIB) and dementia assessments to explore the relationship of microglial activation with underlying pathology in AD and MCI. One of these studies showed increased PK11195 binding in AD patients in comparison with normal controls in parietotemporal regions and a negative correlation with PIB retention levels in the posterior cingulate, a region that also showed lowest glucose metabolism as measured by ^18^F-FDG PET [38]. Another study found increased PK11195 binding in frontal, temporal, parietal, occipital, and cingulate cortices and a twofold elevated PIB retention in the same cortical areas of AD subjects when compared to healthy controls [39]. A study in 14 MCI patients showed that half of them had increased cortical PIB retention while five had increased PK11195 levels, and no regional correlation between the tracers was found [40]. No difference in PK11195 binding between mild and moderate AD patients, MCI patients and control subjects and no regional correlation with PIB retention were found once another study claiming that microglial activation might be associated with later stages of AD alone or that PK11195 might be too insensitive to detect microglial activation at the examined disease stages [41]. This is in disagreement with a study showing increases in microglial activation even during healthy aging [42].

Even if PK11195 is still considered the “gold standard”, the results of PET studies in AD and MCI have been rather discordant, especially in earlier disease stages. New tracers such as *N*-(2,5 dimethoxybenzyl)-*N*-(4-fluoro-2-phenoxyphenyl) acetamide (DAA1106) have higher binding affinity to PBBS and binding characteristics superior to PK11195. One study has so far been conducted in AD showing significantly higher binding in prefrontal, temporal, parietal, occipital, and cingulate cortices, as well as in striatum and cerebellum of AD patients compared with controls [43]. Another PBBS ligand, ^11^C-vinpocetine, has been suggested as potential marker for microgliosis. No difference between AD patients and age-matched control subjects was observed, however, disease and age-specific changes could successfully be displayed [44].

## 4. Conclusions

### 4.1. Clinical Applicability of Microglial Activation Markers

As microglia play a crucial role in the inflammatory response in AD brains, the question arises whether measuring microglial activity in the CSF or brain of AD patients might be useful in clinical routine. In order to serve as a diagnostic tool in AD investigations, these putative microglial biomarkers must be capable of distinguishing between AD patients and healthy individuals as well as from patients suffering from other types of dementia. However, current research suggests that microglial activation markers generally fail to provide high enough diagnostic accuracy to be clinically useful as diagnostic tools on their own. For comparison, the established AD CSF biomarker triad (A*β*42, total-tau and phospho-tau) has very high diagnostic accuracy when evaluated in well-controlled settings, both in cross-sectional studies [45] and in longitudinal studies of early stage patients [46]. Thus, at present, microglial markers are unlikely to add diagnostic performance to the available AD investigation toolbox, although it cannot be excluded that they might be more useful at certain well-defined disease stages [47]. Nonetheless, biomarkers of microglial activation may give clues on underlying pathogenic mechanisms in AD directly *in vivo* in human patients, particularly concerning the ambivalence between neuroprotection and neurotoxicity following microglial activation. In addition, as microglial activation may affect the progression rate in AD, biomarkers could be useful for monitoring of the course of the disease early on.

### 4.2. Further Research on Microglial Activation Markers

Future efforts investigating microglial activation markers may focus on longitudinal studies trying to elucidate whether high levels of microglial activation are beneficial or detrimental in relation to disease progression. Another possible aspect is to ascertain the relation between microglial biomarkers and neuroimaging markers which allow visualizing microglia in the living brain.

### 4.3. Possible Use of Microglial Activation Markers in Future Studies

As microglia could become a target for possible future therapies in AD [48], microglial activation markers may even serve as a measuring tool for evaluating and monitoring the efficiency of these therapeutic interventions. Microglial markers may also be useful to identify subgroups of patients with pronounced alteration of microglial activation, which might be most likely to respond to microglia targeting treatment.
